# Comparative Analysis of Genomic and Transcriptome Sequences Reveals Divergent Patterns of Codon Bias in Wheat and Its Ancestor Species

**DOI:** 10.3389/fgene.2021.732432

**Published:** 2021-08-20

**Authors:** Chenkang Yang, Qi Zhao, Ying Wang, Jiajia Zhao, Ling Qiao, Bangbang Wu, Suxian Yan, Jun Zheng, Xingwei Zheng

**Affiliations:** ^1^School of Life Science, Shanxi University, Taiyuan, China; ^2^State Key Laboratory of Sustainable Dryland Agriculture, Institute of Wheat Research, Shanxi Agricultural University, Linfen, China

**Keywords:** codon usage, *Triticum aestivum*, GC content, tRNA, gene function

## Abstract

The synonymous codons usage shows a characteristic pattern of preference in each organism. This codon usage bias is thought to have evolved for efficient protein synthesis. Synonymous codon usage was studied in genes of the hexaploid wheat *Triticum aestivum* (AABBDD) and its progenitor species, *Triticum urartu* (AA), *Aegilops tauschii* (DD), and *Triticum turgidum* (AABB). *Triticum aestivum* exhibited stronger usage bias for G/C-ending codons than did the three progenitor species, and this bias was especially higher compared to *T. turgidum* and *Ae. tauschii*. High GC content is a primary factor influencing codon usage in *T. aestivum*. Neutrality analysis showed a significant positive correlation (*p*<0.001) between GC12 and GC3 in the four species with regression line slopes near zero (0.16–0.20), suggesting that the effect of mutation on codon usage was only 16–20%. The GC3s values of genes were associated with gene length and distribution density within chromosomes. tRNA abundance data indicated that codon preference corresponded to the relative abundance of isoaccepting tRNAs in the four species. Both mutation and selection have affected synonymous codon usage in hexaploid wheat and its progenitor species. GO enrichment showed that GC biased genes were commonly enriched in physiological processes such as photosynthesis and response to acid chemical. In some certain gene families with important functions, the codon usage of small parts of genes has changed during the evolution process of *T. aestivum*.

## Introduction

In protein synthesis, triplet codons in the mRNA are translated into amino acids to form polypeptide chains. From 2 to 6 synonymous codons can be assigned to a specific amino acid, except for methionine and tryptophan, which are encoded only by AUG and UGG, respectively. Such synonymous codons are not used equally but are instead biased to some optimal codon (Sirihongthong et al., 2019). Codon usage bias, also known as codon bias, can vary among species and even within genes of the same organism (Mukhopadhyay et al., 2007; Bali and Bebok, 2015). Codon usage bias reflects a mutation-selection balance, which can be affected by mutation, translational selection, and genetic drift in a population (Shah and Gilchrist, 2011; Duan et al., 2021). Therefore, understanding the codon usage bias can reveal the effects of long-term evolution on plant genomes.

Long-term studies account of the non-random variation in codon usage have shown that it is not simply a neutral process driven by mutational bias and genetic drift. There is substantial evidence that codon usage bias in some organisms is under selection pressure for translational efficiency (Zalucki et al., 2007; Szövényi et al., 2017; LaBella et al., 2019). Theories suggested that the neutralist model and selective model are not mutually exclusive and that codon usage might reflect a balance between mutational and selection effects (Duret and Mouchiroud, 1999). The “selection-mutation-drift” model was proposed to describe this balance. This model proposes that codon usage bias in genes with high expression is primarily the result of selection at the translational level and that in genes with low expression the bias is mainly caused by either mutation or genetic drift with the selective effect being relatively weak (Sharp and Li, 1987; Bulmer, 1991; Mukhopadhyay et al., 2007). Codon usage bias in unicellular organisms, such as *Escherichia coli* and *Saccharyomyces cerevisiae*, is adequately described by the selection-mutation-drift model. In contrast, codon usage bias is more complex in multicellular eukaryotic organisms. Lavner and Kotlar (2005) found codon bias tends to be high in some weakly expressed genes in mammalian eukaryotes. In such genes, non-optimal codons may have been selected to reduce the expression by reducing the elongation rate during gene translation. In mammals and plants, GC-biased gene conversion (gBGC) has also been proposed as a main driving force in the evolution of base composition, especially in grasses such as the genus *Oryza* and in some non-grass monocot species (Muyle et al., 2011; Clément et al., 2014; Mazumdar et al., 2017).

Codon usage bias in evolution research has been reported on model organisms, including *E. coli* (Krisko et al., 2014), *Dengue virus* (Manokaran et al., 2019), *S. cerevisiae*, *Drosophila melanogaster* (Kliman et al., 2003), and humans (Sauna and Kimchi-Sarfaty, 2011), as well as studies assessing codon usage in plant evolution research (Rensing et al., 2005; Liu et al., 2010; Sahoo et al., 2019; Chi et al., 2020). Plant species are broadly diverse in their gene expression, physiology, and stress response in varied environments. Therefore, the knowledge of codon usage and codon-pair context patterns in plants and the underlying evolutionary forces will advance understanding of the molecular mechanisms of environmental adaptation and biological diversity. Shen et al. (2020) analyzed codon usage patterns in eight citrus species based on coding sequence data, drawing the conclusion that few differences in codon features among citrus species and the genomes of citrus species were conserved. Researches showed that *Arabidopsis* genes show relatively little variation in codon usage, whereas an extreme degree of heterogeneity in codon usage patterns within the rice genome, which is highly correlated with differences in GC content between the genes (Wang and Hickey, 2007). Paralogs with high and low GC contents in rice and other cereals were analyzed and provided evidence for selectively driven codon usage, which represented a potential evolutionary process for the origin of genes with a high GC content in *Gramineae* (Guo et al., 2007).

With three closely related A, B, and D genomes, hexaploid wheat (*Triticum aestivum* L.) is the most widely cultivated crop on earth and is a model for allopolyploidy in plants (Zhang et al., 2020). The progenitor species of the A genome is diploid wild einkorn wheat, *Triticum urartu* (AA; Ling et al., 2018). The ancestor of the D genome is the wild diploid grass *Aegilops tauschii* (DD; Jia et al., 2013), which spontaneously hybridized with cultivated tetraploid wheat *Triticum turgidum* (AABB; Avni et al., 2017) resulting in hexaploid wheat (AABBDD; Appels et al., 2018). Genome sequencing and assembly of wheat and its genome donor species have been finished. The aim of the present study is to assess which evolutionary forces have a significant impact on codon usage bias in hexaploid wheat and its genomic donors.

## Materials and Methods

### Sequence Data

The CDS genomic sequences of *T. urartu* accession G1812, wild tetraploid wheat (*T. turgidum*) accession “Zavitan,” *Ae. tauschii* accession AL8/78, and common wheat cultivar “Chinese Spring” were downloaded from the Ensembl Plants.^1^ The CDS sequences were filtered using Python and the following criteria: (1) the gene is a complete CDS, (2) the length of the CDS sequence is more than 300bp, and (3) the CDS begins with ATG and ends with a termination codon (Liu et al., 2020). The leaf transcriptome data in common wheat and its ancestors were collected from the NCBI^2^ under accessions SRR3274670 (*T. urartu*), SRR11292316 (*T. turgidum*), SRR11292312 (*Ae. tauschii*), and SRR9647023 (*T. aestivum*). In addition, the sequences of genes for grain size (GS; Li and Yang, 2017), the CCT gene family (Zheng et al., 2017), and the prolamin genes (Ling et al., 2018) identified from previous studies were used. The collected fasta sequences of genes from different families were aligned to the local protein database of other species using BALSTX, which was downloaded from URGI database^3^ with the expected value (*E*-value) of the lowest. Meantime, the conserved domains of the gene family were identified using HMMER 3.0.

### Codon Bias Analysis

The codon usage of the *T. aestivum* genome and its ancestor species were analyzed using the software program CodonW 1.4.2^4^ (Peden, 2000). The parameters for the codon usage calculation were listed as following: Relative Synonymous Codon Usage (RSCU), Effective Number of Codon (ENC), GC content (GC), codon first and second and the third position for GC content (GC1, GC2, and GC3), the average GC content of the first and second codons (GC12) and the content of each base in the third position of the synonymous codon (A3s, T3s, G3s, and C3s).

(1) RSCU:

Among the synonymous codons, the codon with the larger RSCU value has a higher usage probability (Wright, 1990). RSCU values were estimated using the following formula:

$$RSCU=\frac{x_{ij}}{\sum_{j}^{n_{i}}x_{ij}}$$

(2) ENC:

A value below 35 indicates high codon usage bias and values above 50 indicates low bias (Sharp and Li, 1987). ENC were calculated by the following formula:

$$ENC_{\exp}=2+s+29∕\left(s^{2}+{\left(1-s\right)}^{2}\right)$$

Effective Number of Codon-plot and neutrality plot analysis were used to analyze factors affecting the codon preference of *T. aestivum* and its ancestor species. In ENC-plot analysis, when ENC value is within the allowable range of the standard curve, the codon preference is caused by base mutation, otherwise the codon preference is caused by natural selection (Cai et al., 2006).

Neutral plot analysis was used to investigate the effect of mutational and selection pressure on the codon usage pattern. The neutrality plot was mapped with the scatterplot to analyze the correlation between GC12 and GC3. When the regression coefficient was close to 0, the correlation was low, indicating that the GC composition for the three positions of the codon differed, the GC content is highly conserved, and the codon preference for the species is mainly affected by natural selection (Su et al., 2009; Nasrullah et al., 2015). Otherwise, the codon bias of species is more affected by mutations.

PR2-plot was used to analyze whether the third base of a codon is biased according to the bias rule (Sueoka, 2001). The bias rule states that if A=T and G=C between the two complementary strands of DNA then there is no mutation and selection bias. If otherwise, then the codon is biased.

### Correspondence Analysis and Determination of Codon

Correspondence analysis (COA) was generated to analyze codon usage changes in different genes. Correlation analysis is used to determine the main factors affecting codon bias (Jia et al., 2015). In COA, all the genes were distributed in multi-dimensional vector spaces using the RSCU COA function of codonW. The first axis represented the most different codon usage changes with sequential decreases represented in the second, third, and fourth axes.

To determine optimal codons of *T. aestivum* genome and its ancestor species, genes with the 5% highest and lowest ENC values were screened to establish a low preference group and a high preference group. Among genes from two groups, the RSUC difference was greater than 0.08, and the codon with the RSUC value greater than 1 was determined to be the optimal codon (Liu et al., 2020).

### Major Factors of Variations in Synonymous Codon Usages Analysis

To determine the factors affecting the codon bias of the *T. aestivum* genome and its ancestor species, we analyzed the number of tRNA anti-codons and the length of genes. Firstly, CDS sequences of common wheat and its ancestor species were divided into four length categories: <1,000bp, 1,000–2,000bp, 2,000–3,000bp, and >3,000bp. Secondly, codon bias analysis for genes of different length was done using CodonW 1.4.2 and the numbers of genes and values of GC3s were calculated. Anti-codons of common wheat and its ancestor species were identified using tRNAscan-SE 2.0 (Chan and Lowe, 2019).

### Gene Function

The rawdata of common wheat and its ancestors were used for quality control using fastp (Chen et al., 2018). To analyze the relationship between codon preference and function of expressed genes, the TPM of CDS sequences from common wheat and its ancestors were calculated using Salmon v1.3.0 (Patro et al., 2017). To determine differences in the function of genes with different codon bias, gene ontogeny (GO) enrichment were conducted separately using genes with different codon bias. For gene annotation, coding sequence regions of each gene in wheat and its ancestor species reference genomes were compared against the default database using eggNOG-mapper (Huerta-Cepas et al., 2017, 2019). The CDS sequences of the TPM>1 of all species were divided into high GC bias genes and high AT bias genes according to GC content of each species. The CDS sequences with different codon bias were calculated *via* GO enrichment analysis using the clusterProfiler R package (Yu et al., 2012).

## Results

### Effective Number of Codons Value and GC Contents

The codon usage characteristics of the *T. aestivum* genome and those of its ancestor species, *T. urartu*, *T. turgidum*, and *Ae. tauschii*, were investigated using CodonW1.4.2. *Triticum aestivum* showed a stronger codon usage bias (ENC 48.6) compared to its ancestor species *T. turgidum* (ENC 51.6) and *Ae. tauschii* (ENC 51.2; Supplementary Table 1). ENC values of the coding genes in the A and B genomes were compared. Among the 57,979 coding genes from the A genome of *T. turgidum*, 3,874 sequences (6.68%) showed high codon usage bias with ENC values less than 35. In genes from the A genome of *T. aestivum*, there were 5,606 sequences (13.66%) with ENC values less than 35. Sequences with ENC values less than 35 accounted for 6.10% of the *T. turgidum* B genome and 12.53% of the *T. aestivum* B genome. Thus, both the A and B genome from *T. aestivum* showed more codon usage bias than the A and B genome from *T. turgidum*.

**Figure 1:**
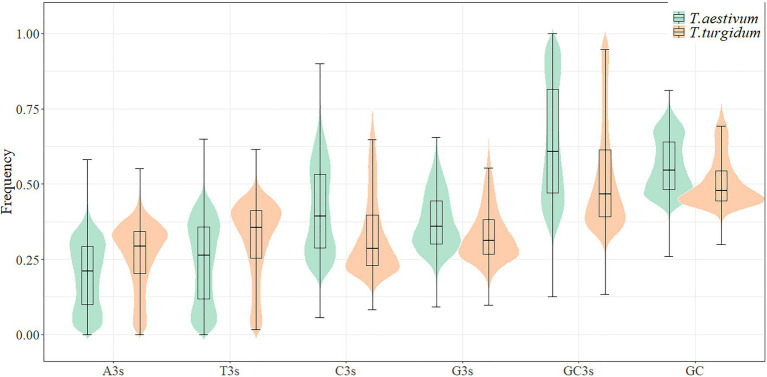
Comparison of base composition between *Triticum aestivum* and *Triticum turgidum*.

GC3s frequencies in *T. aestivum* (63.7%) and *T. urartu* (62.0%) were higher than those in *T. turgidum* (52.6%) and *Ae. tauschii* (54.6%; Figure 1; Supplementary Table 1). Codon usage bias has been shown to correlate with GC composition (Wan et al., 2004). The mean GC contents were examined for the whole coding sequences of the four species and the higher GC content partly explained the bias for G/C-ending codons seen in *T. aestivum* and *T. urartu*. PR2-bias plot analysis showed a non-proportional use of A/T and C/G in the four genomes, with T3>A3 and C3>G3 (Supplementary Figure 1). Neutrality plot results showed that the correlation between GC12 and GC3 was statistically significant (*p*<0.001) in *T. aestivum* (*r*=0.650), *Ae. tauschii* (*r*=0.596), *T. turgidum* (*r*=0.551), and *T. urartu* (*r*=0.517). The slopes of the regression line were close to zero (0.16–0.20, Supplementary Figure 2), indicating that selective pressure should be the dominant factor shaping codon usage pattern in *T. aestivum* and its ancestor species.

### Correspondence Analysis

**Figure 2:**
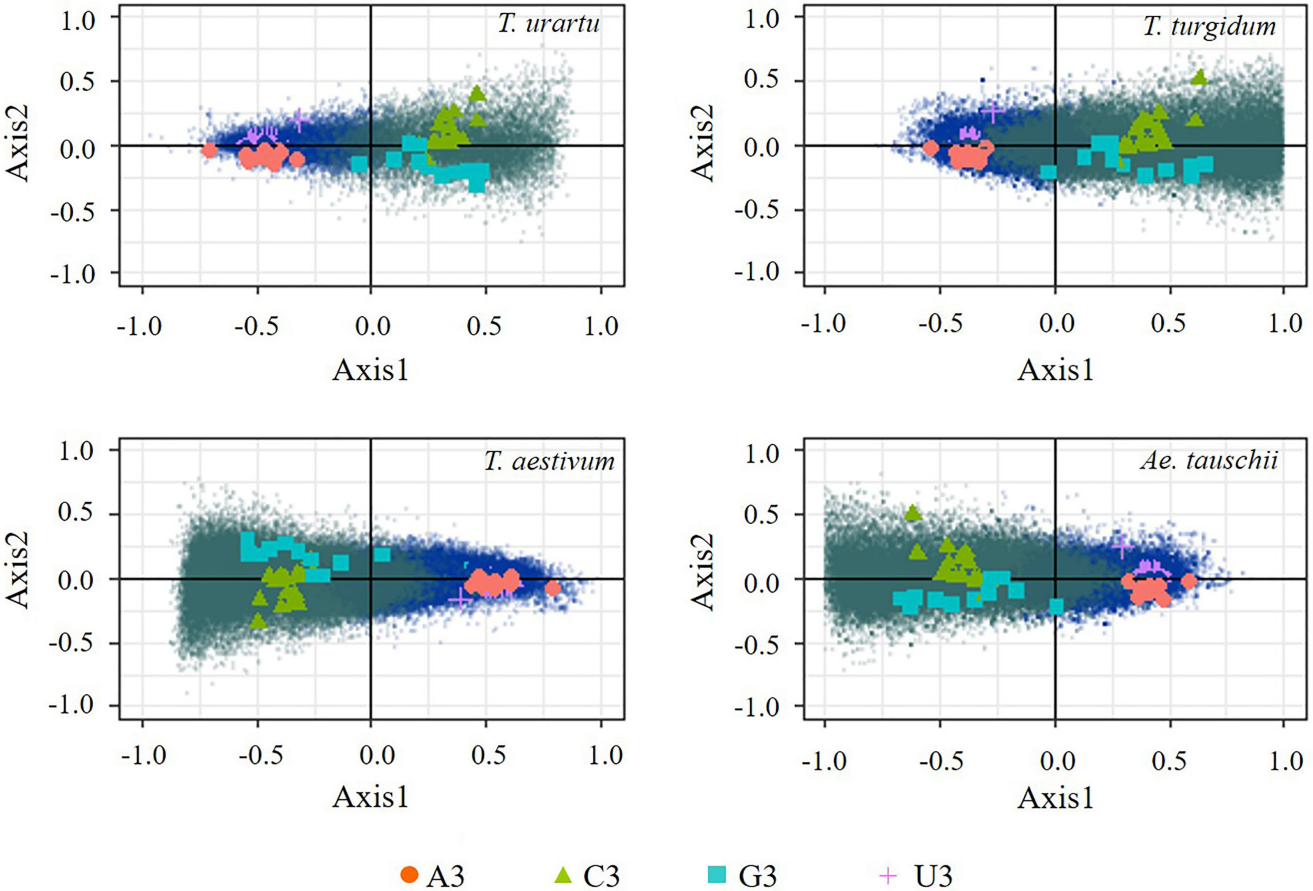
Correspondence analysis based on Relative Synonymous Codon Usage (RSCU) of genes and codons. Blue dots represent genes of AT preference and green dots represent genes of GC preference.

In the present study, COA based on RSCU was expected to further identify the major factors affecting codon usage frequencies and synonymous codon preferences observed in the coding sequences of the four genomes. Genes from the four species were widely distributed along the two explanatory axes, suggesting a strong codon usage bias in these coding sequences (Figure 2). The first and second explanatory axis accounted for 40.2/4.3, 39.1/3.6, 47.9/4.1, and 40.6%/3.7% in *T. urartu*, *T. turgidum*, *T. aestivum*, and *Ae. tauschii*, respectively. Thus, the first axis reflects the primary factor that explains the differences in codon usage among all genes and genes with high GC and high AT were separated along this axis. The corresponding distribution of synonymous codons shows the separation of C/G-ending codons (except AGG that encodes arginine) and A/U-ending codons along this same axis. High G/C genes from *T. urartu* and *T. turgidum* were distributed on the right side of axis 1. The separation of genes on the second axis appears to be largely due to frequency differences in C-ending and G-ending codons among the GC rich genes; however, genes with more A-ending and T-ending codons tended to cluster at the center of axis 2. Correlation analysis showed that the third base preference of coding genes positioned on axis 1 of the COA were significantly related (Supplementary Table 2), indicating that base composition is the main factor affecting codon usage and the COA could be further used in optimal codon identification.

The optimal codons of common wheat and its ancestor species were identified based on the RSCU value (Supplementary Table 3) and the number of optimal codons in *T. urartu*, *T. turgidum*, *T. aestivum*, and *Ae. tauschii* were 21, 14, 22, and 16, respectively. All the optimal codons showed G/C at the third codon position, indicating that wheat and its ancestor species have similar optimal codon preferences for codons with G/C-endings instead of A/U-endings.

### Variations in Synonymous Codon Usages in *Triticum* Genomes

**Figure 3:**
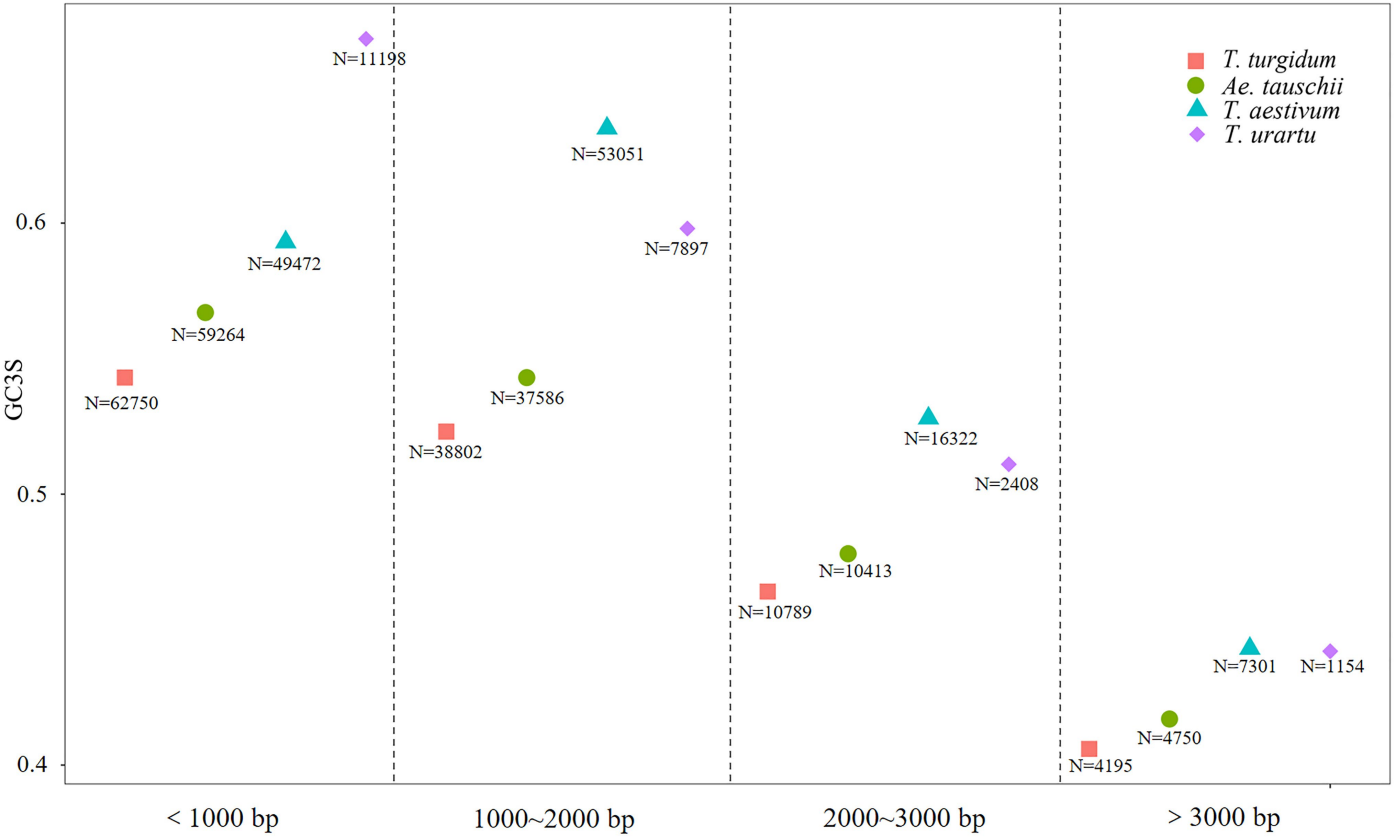
GC3s values in coding sequences from *Triticum urartu*, *Triticum turgidum*, *Triticum aestivum*, and *Aegilops tauschii*. *N* is the number of coding sequences (CDS).

To investigate the effect of coding sequences length on codon usage, coding sequences were classified into four groups: <1,000bp, 1,000–2,000bp, 2,000–3,000bp, and >3,000bp, and the average GC3s values computed for genes from each species (Figure 3). Bias for G/C-ending synonymous codon usage was much stronger in genes coding for short vs. long proteins (Supplementary Table 4). Among coding sequences longer than 1,000bp, more GC-ending synonymous codons were observed in *T. aestivum* than in its ancestor species. However, among sequences <1,000bp, *T. urartu* sequences showed the greatest bias for GC-ending codons. Thus, in longer genes *T. aestivum* showed the greatest tendency to use G/C at the third codon position.

**Figure 4:**
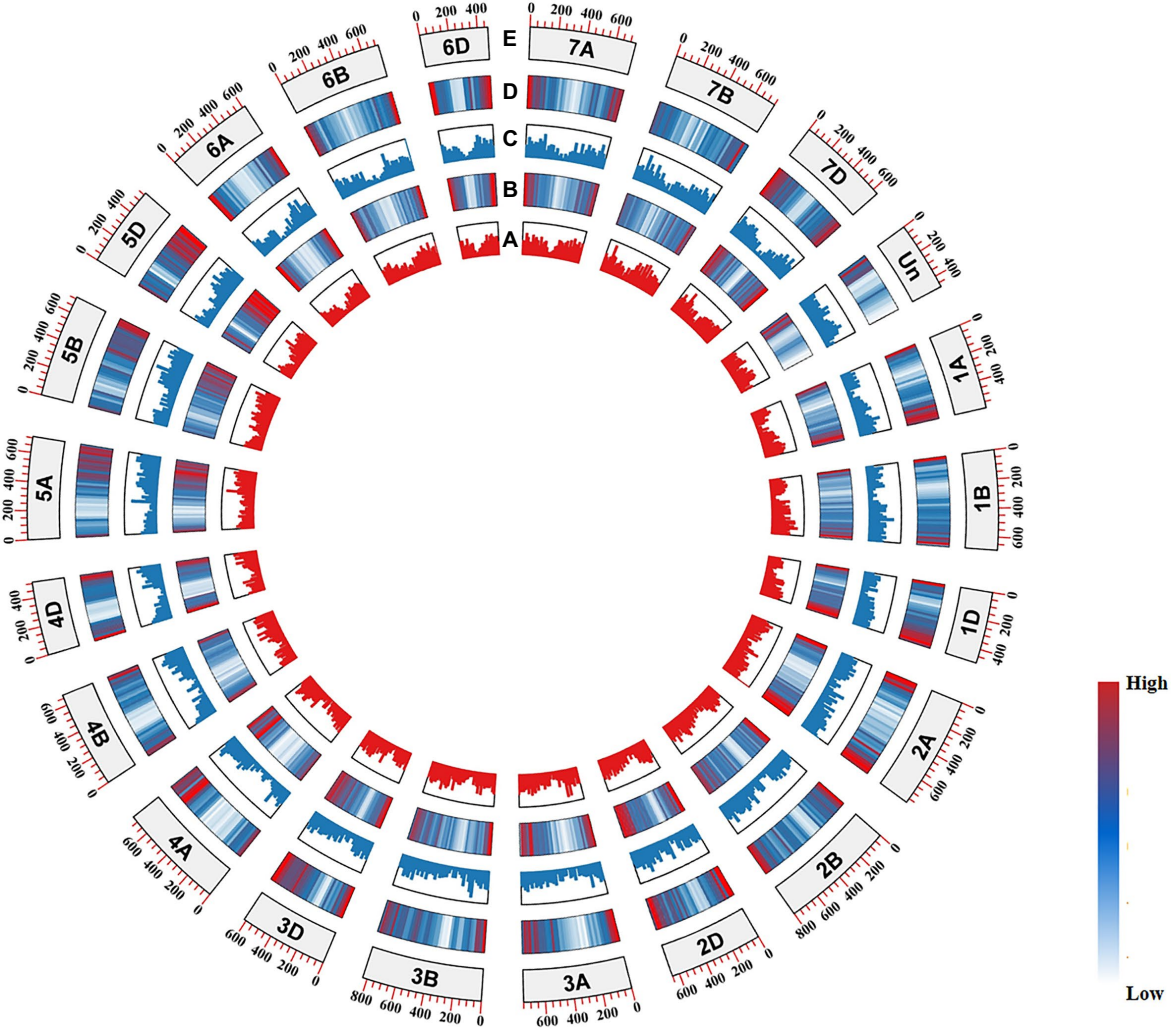
CIRCOS visualization of various *Triticum aestivum* genome data. **(A)** Median GC3 values for genes from the (+) strand. **(B)** Gene density of the (+) strand. **(C)** Median CG3 values for genes from the (−) strand. **(D)** Gene density of the (−) strand. **(E)** Chromosome name and size.

In humans, GC distribution and codon preference have been proved to be related to gene density (Versteeg et al., 2003). In the present study, substantially higher gene density occurred in the distal regions of chromosome arms and genes distributed in these regions exhibited higher GC3s values. In contrast, the centromere regions showed lower gene density and the GC3s values of genes in these regions were low (Figure 4).

### Corresponding tRNA Abundance

**Figure 5:**
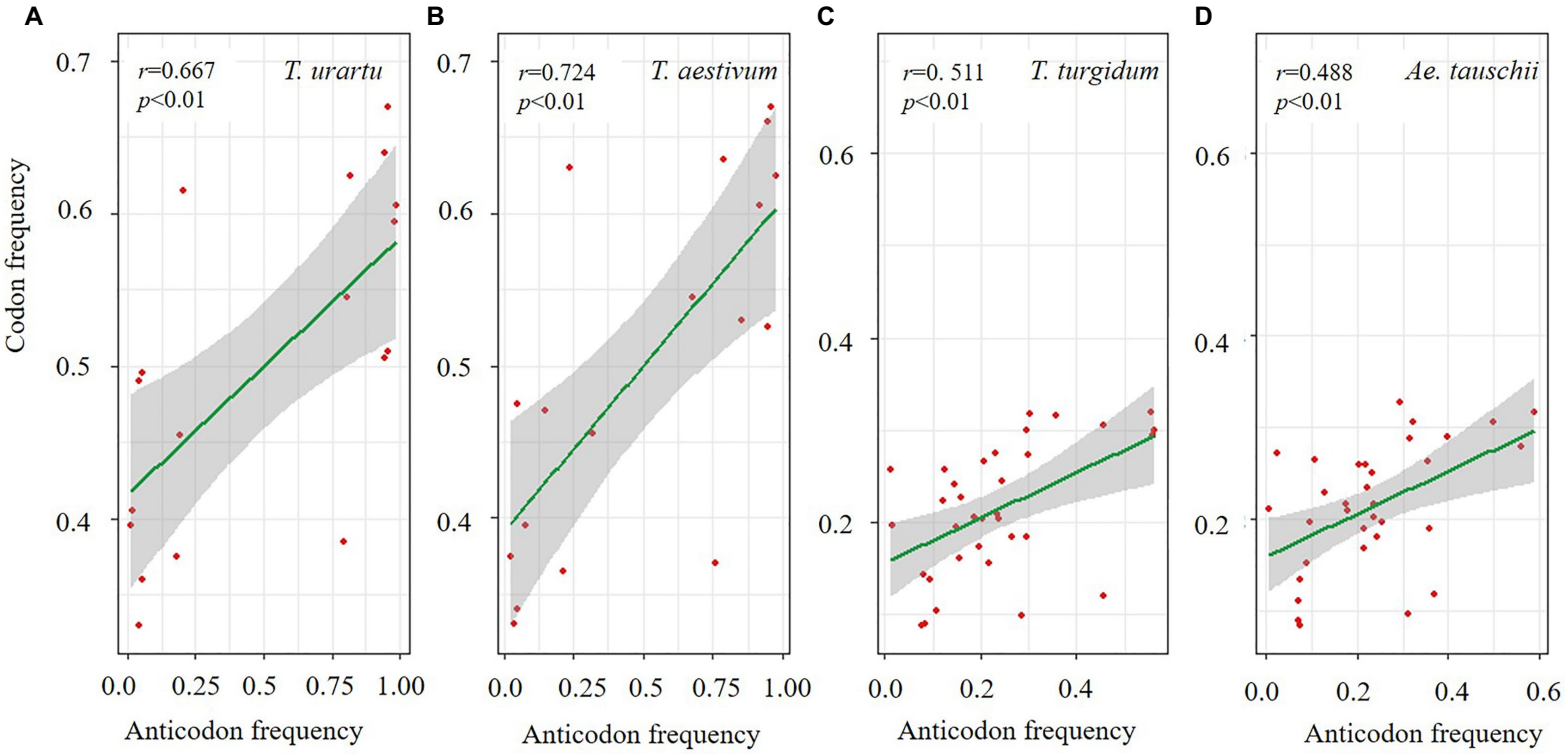
Correlation for common wheat and its three progenitor species between codon and anticodon frequency for amino acids with two codons **(A,B)** and more than four codons **(C,D)**.

Biased usage of synonymous codons can also be constrained by the amount of isoacceptor tRNA to increase translation efficiency (Duret and Mouchiroud, 1999). The frequency of each synonymous codon encoding 21 amino acids was estimated, and we further investigated the amount of corresponding tRNA in the four species (Supplementary Table 5). Significant differences were found among the species for codon frequency and corresponding tRNA abundance. For example, *T. urartu* and *T. aestivum* showed high correlations (*r*=0.667 and 0.724, *p*<0.01) for the amino acids with two synonymous codons (Tyr, Phe, Lys, His, Glu, Gln, Cys, Asp, and Asn; Figures 5A,B). In contrast, amino acid with more than four synonymous codons (Val, Thr, Ser, Pro, Leu, Gly, Arg, and Ala), *T. turgidum* and *Ae. tauschii* showed high correlations (*r*=0.511 and 0.488, *p*<0.01; Figures 5C,D).

### Gene Ontogeny Enrichment of GC/AT Biased Genes

**Figure 6:**
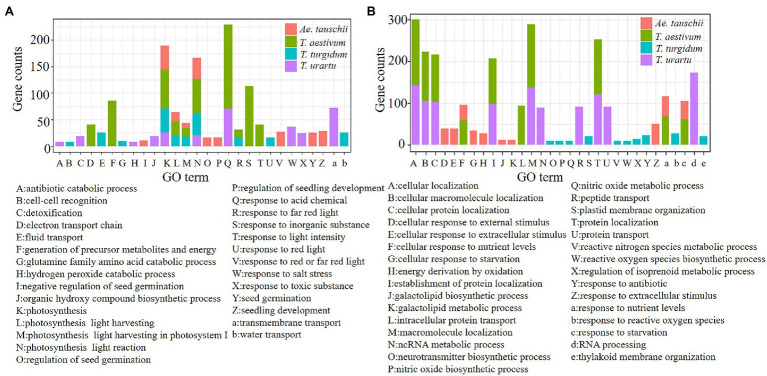
GO enrichment of genes with GC3s **(A)** and AT3s **(B)** biased codons from four species.

Based on GO categories, the functional enrichment of codon usage biased genes from the four species was analyzed, and the top 10 GO terms with values of *p*<0.05 for genes from each species were identified (Figure 6). G/C-ending genes performed different functions compared to A/T-ending genes. The GC3s biased genes from *T. aestivum* and its ancestor species were commonly enriched in genes related to photosynthesis (Figure 6A). Also enriched were transmembrane transport (72/625), and response to acid chemical (71/625) in *T. urartu*, photosynthesis (46/802) and photosynthesis light reaction (43/802) in *T. turgidum*, response to acid chemical (158/1,214) and response to inorganic substance (113/1,214) in *T. aestivum*, and photosynthesis (46/751) and photosynthesis light reaction (40/751) in *Ae. tauschii* (Supplementary Table 6). AT3s biased genes likely perform similar functions in *T. aestivum* and *T. urartu* (Figure 6B) as these genes were enriched in GO terms such as cellular location, macromolecule localization, and protein location. A/T ending genes from *Ae. tauschii* were mainly enriched in response to extracellular stimulus (50/836) and response to nutrient levels (47/836; Supplementary Table 7). The GO enrichment (Supplementary Tables 8 and 9) demonstrated that the evolutionary features reliably reflect the relative function of different codon biased genes.

### Codon Preference in Important Functional Gene Families

The present study shows that synonymous codons had bias at the whole genome level in *T. aestivum* and its ancestor species. We next sought to investigate if codon usage preference changed during evolution for gene families with important functions, including grain size (GS) genes, the CCT gene family regulating flowering, and the quality related prolamin gene family. Based on 36 grain size related genes identified in previous studies in *Triticum* (Li and Yang, 2017), similar GS genes from the A genome of *T. aestivum*, *T. turgidum*, and *T. urartu* were isolated and the GC3s variation of GS genes during polyploidization was calculated (Supplementary Table 10). A difference in the GC3s value between the homologous genes of >0.1 was classified as being codon biased. *Triticum urartu* is the progenitor of the A subgenome of tetraploid *T. turgidum* and hexaploid *T. aestivum* (Ling et al., 2018). From *T. urartu* to *T. aestivum*, seven GS genes were identified with different preferences in codon usage patterns (Table 1). Five of the seven genes, *TRIUR3_05970-T1*, *TRIUR3_33526-T1*, *TRIUR3_34310-T1*, *TRIUR3_08952-T1*, and *TRIUR3_09477-T1*, showed codon usage bias from the time *T. urartu* evolved into *T. turgidum* (Table 2).

**Table 1:** The GC3s variation of grain size genes during polyploidization with *Triticum aestivum* and *Triticum urartu*.

*Triticum aestivum* GS genes	*Triticum urartu* GS genes	GC3s of *Triticum aestivum*	GC3s of *Triticum urartu*	Difference of GC3s
*TraesCS1A02G083000.1*	*TRIUR3_14996-T1*	0.841	0.524	0.317
*TraesCS4A02G012100.1*	*TRIUR3_05970-T1*	0.975	0.793	0.182
*TraesCS2A02G013200.1*	*TRIUR3_33526-T1*	0.756	0.654	0.102
*TraesCS5A02G215100.1*	*TRIUR3_34310-T1*	0.766	0.953	−0.187
*TraesCS7A02G120000.1*	*TRIUR3_08952-T1*	0.603	0.340	0.263
*TraesCS7A02G246500.1*	*TRIUR3_09477-T1*	0.779	0.536	0.243
*TraesCS4A02G430600.1*	*TRIUR3_35245-T1*	0.963	0.566	0.397

**Table 2:** The GC3s variation of grain size genes during polyploidization with *Triticum turgidum* and *Triticum urartu*.

*Triticum turgidum* GS genes	*Triticum urartu* GS genes	GC3s of *Triticum turgidum*	GC3s of *Triticum urartu*	Difference of GC3s
*TRIDC4AG041610.1*	*TRIUR3_05970-T1*	0.911	0.793	0.118
*TRIDC6AG039380.2*	*TRIUR3_33526-T1*	0.784	0.654	0.130
*TRIDC5AG033880.1*	*TRIUR3_34310-T1*	0.763	0.953	−0.190
*TRIDC7BG002860.6*	*TRIUR3_08952-T1*	0.512	0.340	0.172
*TRIDC7AG069150.1*	*TRIUR3_09477-T1*	0.911	0.536	0.375
*TRIDC7AG067090.1*	*TRIUR3_33769-T1*	0.910	0.441	0.469
*TRIDC7AG065170.7*	*TRIUR3_09129-T1*	0.830	0.655	0.175

**Figure 7:**
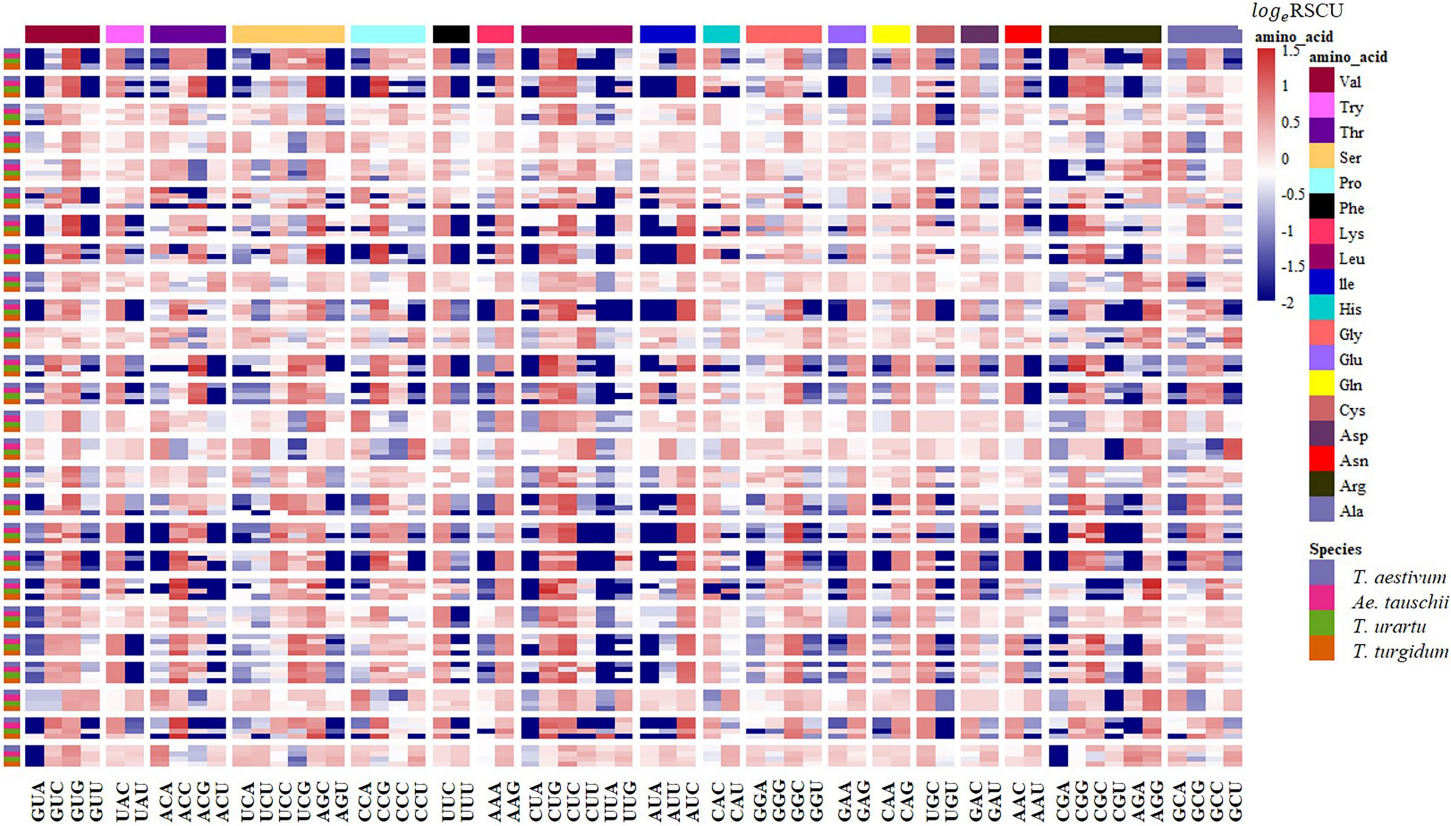
RSCU analysis showing synonymous codon usage preference of the CCT gene families in common wheat and its ancestor species. Columns correspond to the 59 nondegenerate, non-stop codons. Rows correspond to special family genes in the four species. Blue cells indicate low bias codons and red cells indicate high bias codons.

**Figure 8:**
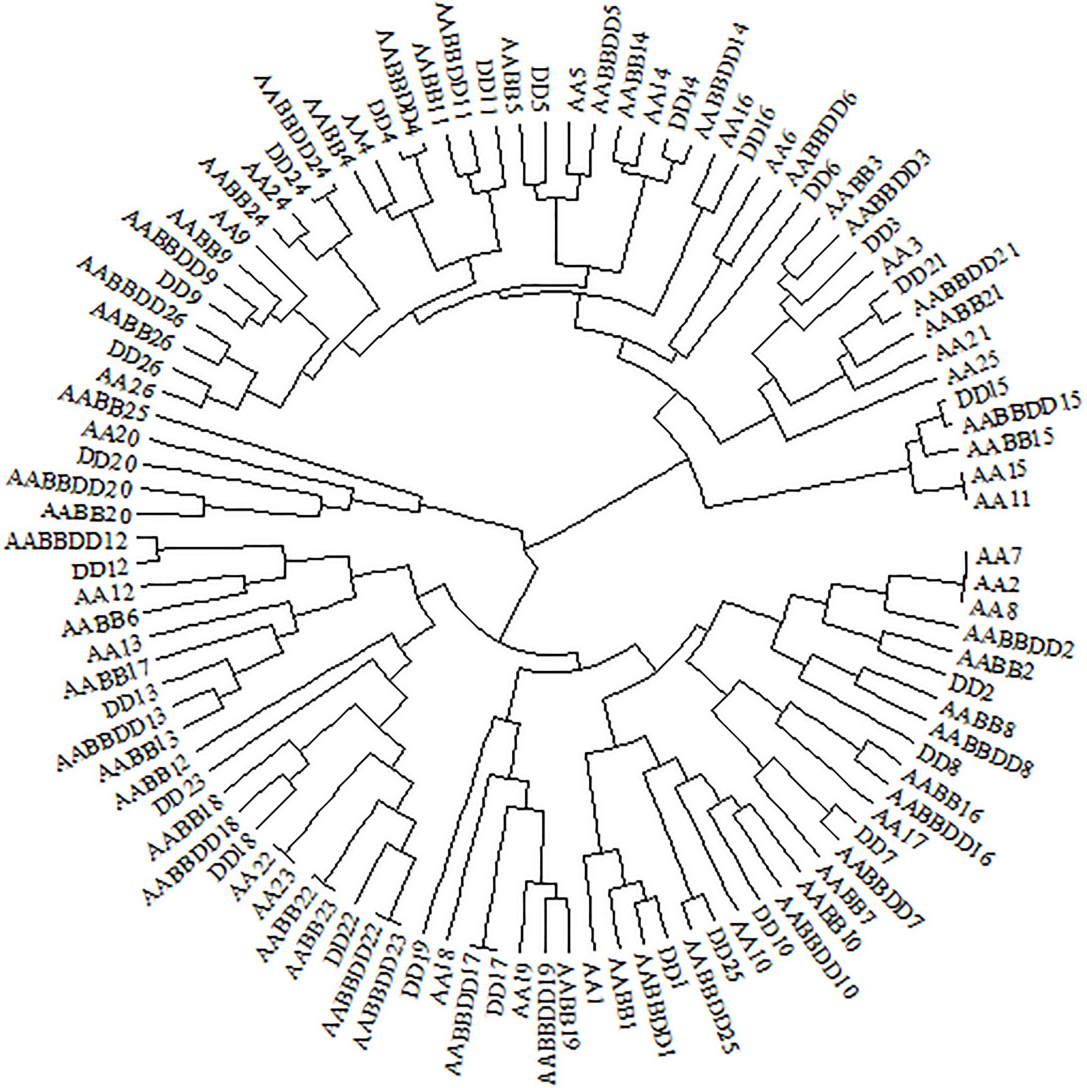
Clustering tree of CCT genes from *Triticum aestivum* (AABBDD), *Aegilops tauschii* (DD), *Triticum turgidum* (AABB), and *Triticum urartu* (AA) according to RSUC values for 59 synonymous codons.

**Figure 9:**
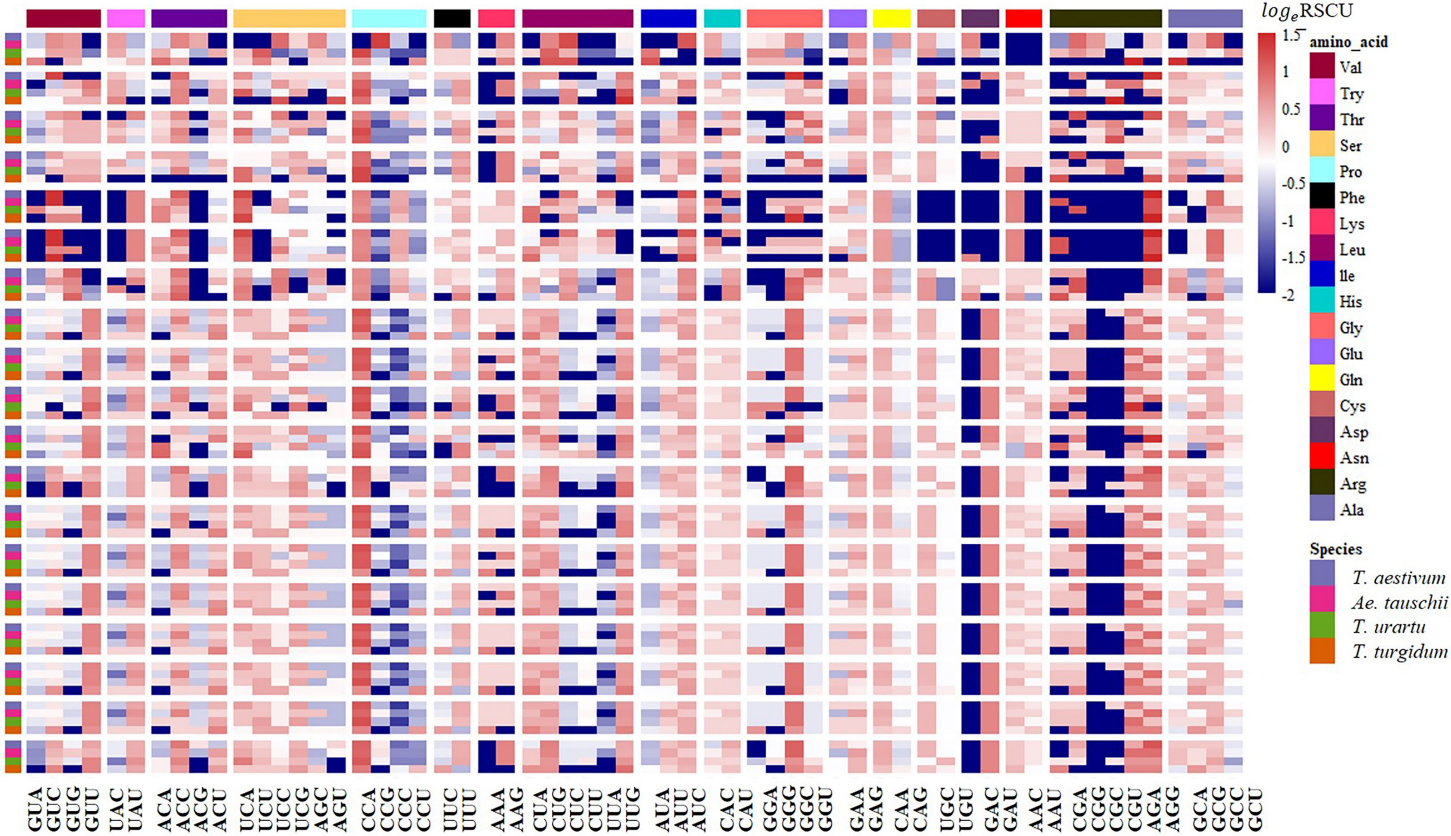
RSCU analysis showing synonymous codon usage preference of the Prolamin gene families in common wheat and its ancestor species. Columns correspond to the 59 nondegenerate, non-stop codons. Rows correspond to special family genes in the four species. Blue cells indicate low bias codons and red cells indicate high bias codons.

The RSCU of each codon was measured for CCT and prolamin genes to investigate the variation in codon usage bias of these genes across the four genomes. Consistent with the results at the genome level, the G/C-ending codons in most of the CCT family genes were overrepresented (RSCU>1), except *CCT24* which showed obvious A/U bias in the third position of synonymous codons. Among the species, the codon usage in *T. aestivum* CCT genes was most like that for genes in the D genome ancestor *Ae. tauschii* (Figure 7). Euclidean clustering of RSCU for codons in each gene showed that among the 26 CCT genes in *T. aestivum*, 15 were like *Ae. tauschii*, eight were like *T. turgidum*, and the last three were like *T. urartu* (Figure 8). For the prolamin genes, some optimal codons identified at the genome level (Supplementary Table 5) were underrepresented (RSCU<1) including GUG encoding Val, UAC encoding Try, CCG encoding Pro, UUC encoding Phe, CUC encoding Leu, CAG encoding Glu, GAC encoding Asp, and CGC encoding Arg (Figure 9).

## Discussion

*Triticum aestivum* has a close phylogenetic relationship with its ancestor species. However, due to the long-term evolutionary processes and the various environments, where the plants evolved, the codon usage patterns among the species differ. In the present study, we analyzed the codon bias patterns in *T. aestivum* and its progenitor species and found that *T. aestivum* has similar preference in codon usage with *T. urartu* and that it differs most from *T. turgidum*. This phenomenon is mainly explained by the similar genomic GC content in *T. aestivum* and *T. urartu* (Supplementary Table 1), which considered to be the strongest determinant of codon usage variation caused by mutational processes across species (Muyle et al., 2011; Plotkin and Kudla, 2011).

### tRNA Abundance Supports the Role of Selection for Translational Efficiency

Codon bias is determined by a balance between mutation, drift, and selection for optimal translational efficiency and/or accuracy (De La Torre et al., 2015). In many cases, codon usage correlates with the cognate tRNA abundance with highly used codons generally having higher intracellular tRNA concentrations than low-use codons (Zalucki et al., 2009). This fact indicates that tRNA abundance may be the main selection pressure for synonymous codon usage (Mohanta et al., 2020). In the present study of *Triticum*, more codon-anticodon interaction occurred in amino acids with two synonymous codons in *T. aestivum* and *T. urartu* and in amino acids with four or six synonymous codons in *T. turgidum* and *Ae. tauschii*. This result also provides evidence for the similar codon usage bias in *T. aestivum* and *T. urartu*. In contrast, for codons encoding glutamine from *T. aestivum* and *T. urartu*, corresponding tRNA copy numbers for the optimal codon (CAG) were much less than for the non-optimal codon (CAA), suggesting that the primary factor influencing codon-usage for these amino acids is not translation efficiency (Kanaya et al., 2001). Simultaneously, we considered the influence of codon swinging on tRNA abundance and codon usage preference. Crick (1966) proposed the wobble hypothesis based on stereochemistry to explain this phenomenon: when the first anticodon is A or C, only one codon can be recognized; when the first anticodon is G or U, two codons can be recognized, where G can identify C and U, where U can identify A and G. Due to the wobble phenomenon, a tRNA anticodon can be combined with more than one mRNA codon. We conjecture that UUG oscillates when recognizing CAA, so that more UUG can recognize the optimal codon CAG. We also found the same phenomenon in Ser. Wobble rules say that tRNAs with a GCU anticodon can pair with AGU, which explains the apparent discrepancy in the ability to translate AGU codons to Ser with extremely few tRNAs with ACU anticodons (Supplementary Table 5). In a recent research of 128 species of the plants (Mohanta et al., 2020), high and low frequencies of wobble base-pairing were occurred at the G:U base pairing to meet translational demand.

### *Triticum aestivum* Showed More GC3 Codon Bias in Genes With Optimal Length

Duret and Mouchiroud (1999) found that codon usage bias is negatively correlated with protein length in *Caenorhabditis elegans*, *D. melanogaster*, and *Arabidopsis thaliana*. Gene length showed a negative correlation with GC3 content in non-grass monocot species (Mazumdar et al., 2017). The same phenomenon was found in the three progenitor species, as coding sequences shorter than 1,000bp had the highest GC3 codon bias (Figure 3). In *T. aestivum*, though, synonymous codon bias increased among coding sequences of 1,000–2,000bp compared to short sequences (<1,000bp). In the *T. aestivum* genome, we found that most coding sequences were between 1,000 and 2,000bp (*N*=53, 051), which is consistent with the result for *T. aestivum* transcript sequences reported by Wang et al. (2019). All the optimal codons identified in the *Triticeae* in the present study preferred G/C-endings at the genome wide level, and the GC3 content of coding sequences shorter than 2,000bp was higher than that for longer genes. This result supports the idea that in *T. aestivum* sequences of less than 2,000bp are the optimal length for translation, especially for important functional genes, and that the use of optimal codons increases their translational efficiency and accuracy. The optimal gene length of *T. aestivum* may be longer than that of its ancestral species, and perhaps the process of evolving into a complex hexaploid species enabled *T. aestivum* to translate longer proteins with minimal mismatches.

### Particular Gene Families Showed Different Codon Usage Preference Compared With the Whole Genome

Genes for particular physiological processes, such as photosynthesis, response to salt stress/acid chemical/cold stress, and seed germination, were biased to GC-ending codons. In contrast, A/U-ending biased genes tended to be associated with the function of basic biological processes, like RNA processing, protein localization, protein transport, and membrane organization (Supplementary Tables 8 and 9). Some important genes always maintained conserved codon usage bias. For example, we identified 1,755 disease resistance genes in *Ae. tauschii* (Supplementary Table 11; Jia et al., 2013) and compared the GC3s contents with the corresponding homoeologous genes in D genome of *T. aestivum*. Only 19 genes had a GC3 difference of more than 0.1, an exceptionally low level (Supplementary Table 12).

CCT family genes are important for the flowering process, which enables the adaptation for reproductive success in various geographical environments (Yang et al., 2013). RSCU values of 59 synonymous codons in the CCT gene family from the four species were investigated and most CCT genes from *T. aestivum* (15/26) were clustered together with genes from *Ae. tauschii*, indicating that CCT genes from *T. aestivum* and *Ae. tauschii* were similar in codon usage frequency. We further measured the GC3 values of CCT genes and found that *T. aestivum* and *Ae. tauschii* CCTs showed similar G/C preference at the third position of codons (Supplementary Figure 3A; Supplementary Table 13), which was consistent with the RSCU results. Previous studies showed that the rapid evolution of the CCT gene family promotes the adaptability of *Ae. tauschii* (Zheng et al., 2017), the similar codon usage bias implies that CCT genes from *T. aestivum* maintained the functions from its D progenitor, *Ae. tauschii*, presumably to enhance environment adaptability. The prolamin sequences of the four species are highly similar, but there are obvious differences in the usage of codons, especially the optimal codons (Supplementary Figure 3B; Supplementary Table 14). As a donor of the D genome of wheat, *Ae. tauschii* provided wheat with better adaptability and quality traits (Jia et al., 2013).

For codon optimization, not only must the codon preference and tRNA abundance be considered, but also other factors including local mRNA folding, codon coordination, and codon correlation. By both natural evolution and genetic engineering, these properties can be altered to gain additional transcriptional regulatory ability for appropriate gene expression under specific cell conditions.

## Data Availability Statement

The datasets presented in this study can be found in online repositories. The names of the repository/repositories and accession number(s) can be found in the article/Supplementary Material.

## Author Contributions

CY collected datasets and performed the bioinformatics analysis. CY, QZ, and YW wrote original draft. XZ and JuZ conducted funding acquisition, project administration, and manuscript review and editing. JiZ and LQ plotted the figures. BW and SY prepared the supplementary data. All authors contributed to the article and approved the submitted version.

## Conflict of Interest

The authors declare that the research was conducted in the absence of any commercial or financial relationships that could be construed as a potential conflict of interest.

## Publisher’s Note

All claims expressed in this article are solely those of the authors and do not necessarily represent those of their affiliated organizations, or those of the publisher, the editors and the reviewers. Any product that may be evaluated in this article, or claim that may be made by its manufacturer, is not guaranteed or endorsed by the publisher.

## Footnotes

^1^ http://plants.ensembl.org/index.html

^2^ https://www.ncbi.nlm.nih.gov/

^3^ https://urgi.versailles.inra.fr/download/iwgsc/

^4^ http://codonw.sourceforge.net/

## References

[ref1] Appels R. Eversole K. Stein N. Feuillet C. Keller B. Rogers J.. (2018). Shifting the limits in wheat research and breeding using a fully annotated reference genome. Science 361:eaar7191. 10.1126/science.aar7191, PMID: 30115783

[ref2] Avni R. Nave M. Barad O. Baruch K. Twardziok S. O. Gundlach H.. (2017). Wild emmer genome architecture and diversity elucidate wheat evolution and domestication. Science 357, 93–97. 10.1126/science.aan0032, PMID: 28684525

[ref3] Bali V. Bebok Z. (2015). Decoding mechanisms by which silent codon changes influence protein biogenesis and function. Int. J. Biochem. Cell B 64, 58–74. 10.1016/j.biocel.2015.03.011, PMID: 25817479 PMC4461553

[ref4] Bulmer M. (1991). The selection-mutation-drift theory of synonymous codon usage. Genetics 129, 897–907. 10.1093/genetics/129.3.897, PMID: 1752426 PMC1204756

[ref5] Cai Z. Q. Penaflor C. Kuehl J. V. Leebens-Mack J. Carlson J. E. dePamphilis C. W.. (2006). Complete plastid genome sequences of Drimys, Liriodendron, and Piper: implications for the phylogenetic relationships of magnoliids. BMC Evol. Biol. 6:77. 10.1186/1471-2148-6-77, PMID: 17020608 PMC1626487

[ref6] Chan P. P. Lowe T. M. (2019). tRNAscan-SE: searching for tRNA genes in genomic sequences. Methods Mol. Biol. 1962, 1–14. 10.1007/978-1-4939-9173-01, PMID: 31020551 PMC6768409

[ref7] Chen S. Zhou Y. Chen Y. Gu J. (2018). fastp: an ultra-fast all-in-one FASTQ preprocessor. Bioinformatics 34, i884–i890. 10.1093/bioinformatics/bty560, PMID: 30423086 PMC6129281

[ref8] Chi X. Zhang F. Dong Q. Chen S. (2020). Insights into comparative genomics, codon usage bias, and phylogenetic relationship of species from *Biebersteiniaceae* and *Nitrariaceae* based on complete chloroplast genomes. Plants 9:1605. 10.3390/plants9111605, PMID: 33218207 PMC7699153

[ref9] Clément Y. Fustier M. A. Nabholz B. Glémin S. (2014). The bimodal distribution of genic GC content is ancestral to monocot species. Genome Biol. Evol. 7, 336–348. 10.1093/gbe/evu278, PMID: 25527839 PMC4316631

[ref10] Crick F. H. (1966). Codon--anticodon pairing: the wobble hypothesis. J. Biochem. Mol. Biol. 19, 548–555. 10.1016/s0022-2836(66)80022-0 5969078

[ref11] De La Torre A. R. Lin Y. C. Van de Peer Y. Ingvarsson P. K. (2015). Genome-wide analysis reveals diverged patterns of codon bias, gene expression, and rates of sequence evolution in picea gene families. Genome Biol. Evol. 7, 1002–1015. 10.1093/gbe/evv044, PMID: 25747252 PMC4419791

[ref12] Duan H. Zhang Q. Wang C. Li F. Tian F. Lu Y.. (2021). Analysis of codon usage patterns of the chloroplast genome in *Delphinium grandiflorum* L. reveals a preference for AT-ending codons as a result of major selection constraints. PeerJ 9:e10787. 10.7717/peerj.10787, PMID: 33552742 PMC7819120

[ref13] Duret L. Mouchiroud D. (1999). Expression pattern and, surprisingly, gene length shape codon usage in *Caenorhabditis*, *drosophila*, and *Arabidopsis*. Proc. Natl. Acad. Sci. U. S. A. 96, 4482–4487. 10.1073/pnas.96.8.4482 10200288 PMC16358

[ref14] Guo X. Y. Bao J. D. Fan L. J. (2007). Evidence of selectively driven codon usage in rice: implications for GC content evolution of *Gramineae* genes. FEBS Lett. 581, 1015–1021. 10.1016/j.febslet.2007.01.088, PMID: 17306258

[ref15] Huerta-Cepas J. Forslund K. Coelho L. P. Szklarczyk D. Jensen L. J. von Mering C.. (2017). Fast genome-wide functional annotation through orthology assignment by eggNOG-mapper. Mol. Biol. Evol. 34, 2115–2122. 10.1093/molbev/msx148, PMID: 28460117 PMC5850834

[ref16] Huerta-Cepas J. Szklarczyk D. Heller D. Hernández-Plaza A. Forslund S. K. Cook H.. (2019). eggNOG 5.0: a hierarchical, functionally and phylogenetically annotated orthology resource based on 5090 organisms and 2502 viruses. Nucleic Acids Res. 47, D309–D314. 10.1093/nar/gky1085, PMID: 30418610 PMC6324079

[ref17] Jia X. Liu S. Y. Zheng H. Li B. Qi Q. Wei L.. (2015). Non-uniqueness of factors constraint on the codon usage in *Bombyx mori*. BMC Genomics 16:356. 10.1186/s12864-015-1596-z, PMID: 25943559 PMC4422305

[ref18] Jia J. Z. Zhao S. C. Kong X. Y. Li Y. R. Zhao G. Y. He W. M.. (2013). *Aegilops tauschii* draft genome sequence reveals a gene repertoire for wheat adaptation. Nature 496, 91–95. 10.1038/nature12028, PMID: 23535592

[ref19] Kanaya S. Yamada Y. Kinouchi M. Kudo Y. Ikemura T. (2001). Codon usage and tRNA genes in eukaryotes: correlation of codon usage diversity with translation efficiency and with CG-dinucleotide usage as assessed by multivariate analysis. J. Mol. Evol. 53, 290–298. 10.1007/s002390010219, PMID: 11675589

[ref20] Kliman R. M. Irving N. Santiago M. (2003). Selection conflicts, gene expression, and codon usage trends in yeast. J. Mol. Evol. 57, 98–109. 10.1007/s00239-003-2459-9, PMID: 12962310

[ref21] Krisko A. Copic T. Gabaldón T. Lehner B. Supek F. (2014). Inferring gene function from evolutionary change in signatures of translation efficiency. Genome Biol. 15:R44. 10.1186/gb-2014-15-3-r44, PMID: 24580753 PMC4054840

[ref22] LaBella A. L. Opulente D. A. Steenwyk J. L. Hittinger C. T. Rokas A. (2019). Variation and selection on codon usage bias across an entire subphylum. PLoS Genet. 15:e1008304. 10.1371/journal.pgen.1008304, PMID: 31365533 PMC6701816

[ref23] Lavner Y. Kotlar D. (2005). Codon bias as a factor in regulating expression via translation rate in the human genome. Gene 345, 127–138. 10.1016/j.gene.2004.11.035, PMID: 15716084

[ref24] Li W. L. Yang B. (2017). Translational genomics of grain size regulation in wheat. Theor. Appl. Genet. 130, 1765–1771. 10.1007/s00122-017-2953-x, PMID: 28765985

[ref25] Ling H. Q. Ma B. Shi X. L. Liu H. Dong L. L. Sun H.. (2018). Genome sequence of the progenitor of wheat A subgenome *Triticum urartu*. Nature 557, 424–428. 10.1038/s41586-018-0108-0, PMID: 29743678 PMC6784869

[ref26] Liu H. He R. Zhang H. Huang Y. Tian M. Zhang J. (2010). Analysis of synonymous codon usage in Zea mays. Mol. Biol. Rep. 37, 677–684. 10.1007/s11033-009-9521-7, PMID: 19330534

[ref27] Liu X. Y. Li Y. Ji K. K. Zhu J. Ling P. Zhou T.. (2020). Genome-wide codon usage pattern analysis reveals the correlation between codon usage bias and gene expression in *Cuscuta australis*. Genomics 112, 2695–2702. 10.1016/j.ygeno.2020.03.002, PMID: 32145379

[ref28] Manokaran G. Sujatmoko X. McPherson K. G. Simmons C. P. (2019). Attenuation of a dengue virus replicon by codon deoptimization of nonstructural genes. Vaccine 37, 2857–2863. 10.1016/j.vaccine.2019.03.062, PMID: 31000413

[ref29] Mazumdar P. Binti O. R. Mebus K. Ramakrishnan N. Ann H. J. (2017). Codon usage and codon pair patterns in non-grass monocot genomes. Ann. Bot. 120, 893–909. 10.1093/aob/mcx112, PMID: 29155926 PMC5710610

[ref30] Mohanta T. K. Mishra A. K. Hashem A. Abd Allah E. F. Khan A. L. Al-Harrasi A. (2020). Construction of anti-codon table of the plant kingdom and evolution of tRNA selenocysteine (tRNA^Sec^). BMC Genomics 21:804. 10.1186/s12864-020-07216-3, PMID: 33213362 PMC7678280

[ref31] Mukhopadhyay P. Basak S. Ghosh T. C. (2007). Nature of selective constraints on synonymous codon usage of rice differs in GC-poor and GC-rich genes. Gene 400, 71–81. 10.1016/j.gene.2007.05.027, PMID: 17629420

[ref32] Muyle A. Serres-Giardi L. Ressayre A. Escobar J. Glémin S. (2011). GC-biased gene conversion and selection affect GC content in the *Oryza* genus (rice). Mol. Biol. Evol. 28, 2695–2706. 10.1093/molbev/msr104, PMID: 21504892

[ref33] Nasrullah I. Butt A. M. Tahir S. Idrees M. Tong Y. (2015). Genomic analysis of codon usage shows influence of mutation pressure, natural selection, and host features on Marburg virus evolution. BMC Evol. Biol. 15:174. 10.1186/s12862-015-0456-4, PMID: 26306510 PMC4550055

[ref34] Patro R. Duggal G. Love M. I. Irizarry R. A. Kingsford C. (2017). Salmon provides fast and bias-aware quantification of transcript expression. Nat. Methods 14, 417–419. 10.1038/nmeth.4197, PMID: 28263959 PMC5600148

[ref35] Peden J. F. (2000). Analysis of codon usage. dissertation/master’s thesis. Nottingham: University of Nottingham.

[ref36] Plotkin J. B. Kudla G. (2011). Synonymous but not the same: the causes and consequences of codon bias. Nat. Rev. Genet. 12, 32–42. 10.1038/nrg2899, PMID: 21102527 PMC3074964

[ref37] Rensing S. A. Fritzowsky D. Lang D. Reski R. (2005). Protein encoding genes in an ancient plant: analysis of codon usage, retained genes and splice sites in a moss, *Physcomitrella patens*. BMC Genomics 6:43. 10.1186/1471-2164-6-43, PMID: 15784153 PMC1079823

[ref38] Sahoo S. Das S. S. Rakshit R. (2019). Codon usage pattern and predicted gene expression in *Arabidopsis thaliana*. Gene X 2:100012. 10.1016/j.gene.2019.100012, PMID: 32550546 PMC7286098

[ref39] Sauna Z. E. Kimchi-Sarfaty C. (2011). Understanding the contribution of synonymous mutations to human disease. Nat. Rev. Genet. 12, 683–691. 10.1038/nrg3051, PMID: 21878961

[ref40] Shah P. Gilchrist M. A. (2011). Explaining complex codon usage patterns with selection for translational efficiency, mutation bias, and genetic drift. Proc. Natl. Acad. Sci. U. S. A. 108, 10231–10236. 10.1073/pnas.1016719108, PMID: 21646514 PMC3121864

[ref41] Sharp P. M. Li W. H. (1987). The rate of synonymous substitution in enterobacterial genes is inversely related to codon usage bias. Mol. Biol. Evol. 4, 222–230. 10.1093/oxfordjournals.molbev.a040443, PMID: 3328816

[ref42] Shen Z. Gan Z. Zhang F. Yi X. Zhang J. Wan X. (2020). Analysis of codon usage patterns in citrus based on coding sequence data. BMC Genomics 21(Suppl. 5):234. 10.1186/s12864-020-6641-x, PMID: 33327935 PMC7739459

[ref43] Sirihongthong T. Jitobaom K. Phakaratsakul S. Boonarkart C. Suptawiwat O. Auewarakul P. (2019). The relationship of codon usage to the replication strategy of parvoviruses. Arch. Virol. 164, 2479–2491. 10.1007/s00705-019-04343-5, PMID: 31321584

[ref44] Su M. W. Lin H. M. Yuan H. S. Chu W. C. (2009). Categorizing host-dependent RNA viruses by principal component analysis of their codon usage preferences. J. Comput. Biol. 16, 1539–1547. 10.1089/cmb.2009.0046, PMID: 19958082

[ref45] Sueoka N. (2001). Near homogeneity of PR2-bias fingerprints in the human genome and their implications in phylogenetic analyses. J. Mol. Evol. 53, 469–476. 10.1007/s002390010237, PMID: 11675607

[ref46] Szövényi P. Ullrich K. K. Rensing S. A. Lang D. van Gessel N. Stenøien H. K.. (2017). Selfing in haploid plants and efficacy of selection: codon usage bias in the model moss *Physcomitrella patens*. Genome Biol. Evol. 9, 1528–1546. 10.1093/gbe/evx098, PMID: 28549175 PMC5507605

[ref47] Versteeg R. van Schaik B. D. van Batenburg M. F. Roos M. Monajemi R. Caron H.. (2003). The human transcriptome map reveals extremes in gene density, intron length, GC content, and repeat pattern for domains of highly and weakly expressed genes. Genome Res. 13, 1998–2004. 10.1101/gr.1649303, PMID: 12915492 PMC403669

[ref48] Wan X. F. Xu D. Kleinhofs A. Zhou J. Z. (2004). Quantitative relationship between synonymous codon usage bias and GC composition across unicellular genomes. BMC Evol. Biol. 4:19. 10.1186/1471-2148-4-19, PMID: 15222899 PMC476735

[ref49] Wang X. M. Chen S. Y. Shi X. Liu D. N. Zhao P. Lu Y. Z.. (2019). Hybrid sequencing reveals insight into heat sensing and signaling of bread wheat. Plant J. 98, 1015–1032. 10.1111/tpj.14299, PMID: 30891832 PMC6850178

[ref50] Wang H. C. Hickey D. A. (2007). Rapid divergence of codon usage patterns within the rice genome. BMC Evol. Biol. 7(Suppl. 1):S6. 10.1186/1471-2148-7-S1-S6, PMID: 17288579 PMC1796615

[ref51] Wright F. (1990). The ‘effective number of codons’ used in a gene. Gene 87, 23–29. 10.1016/0378-1119(90)90491-9, PMID: 2110097

[ref52] Yang Q. Li Z. Li W. Q. Ku L. X. Wang C. Ye J. R.. (2013). CACTA-like transposable element in *ZmCCT* attenuated photoperiod sensitivity and accelerated the postdomestication spread of maize. Proc. Natl. Acad. Sci. U. S. A. 110, 16969–16974. 10.1073/pnas.1310949110, PMID: 24089449 PMC3801022

[ref53] Yu G. C. Wang L. G. Han Y. Y. He Q. Y. (2012). clusterProfiler: an R package for comparing biological themes among gene clusters. OMICS 16, 284–287. 10.1089/omi.2011.0118, PMID: 22455463 PMC3339379

[ref54] Zalucki Y. M. Beacham I. R. Jennings M. P. (2009). Biased codon usage in signal peptides: a role in protein export. Trends Microbiol. 17:19307122, 146–150. 10.1016/j.tim.2009.01.005 19307122

[ref55] Zalucki Y. M. Power P. M. Jennings M. P. (2007). Selection for efficient translation initiation biases codon usage at second amino acid position in secretory proteins. Nucleic Acids Res. 35:5748. 10.1093/nar/gkm577, PMID: 17717002 PMC2034453

[ref56] Zhang R. Z. Huang S. Y. Li S. M. Song G. Q. Li Y. L. Li W.. (2020). Evolution of PHAS loci in the young spike of allohexaploid wheat. BMC Genomics 21:200. 10.1186/s12864-020-6582-4, PMID: 32131726 PMC7057497

[ref57] Zheng X. W. Li X. H. Ge C. Chang J. Z. Shi M. M. Chen J. L.. (2017). Characterization of the CCT family and analysis of gene expression in *Aegilops tauschii*. PLoS One 12:e0189333. 10.1371/journal.pone.0189333, PMID: 29220383 PMC5722339

