# Supplementary material for: Comparative Analysis of Genomic and Transcriptome Sequences Reveals Divergent Patterns of Codon Bias in Wheat and Its Ancestor Species
Source: Front Genet. 2021 Aug 20;12:732432. doi: 10.3389/fgene.2021.732432 (PMC8417831; doi:10.3389/fgene.2021.732432)
Supplement: Supplementary file 2 [file Presentation_1.PPTX]

## Slide 1
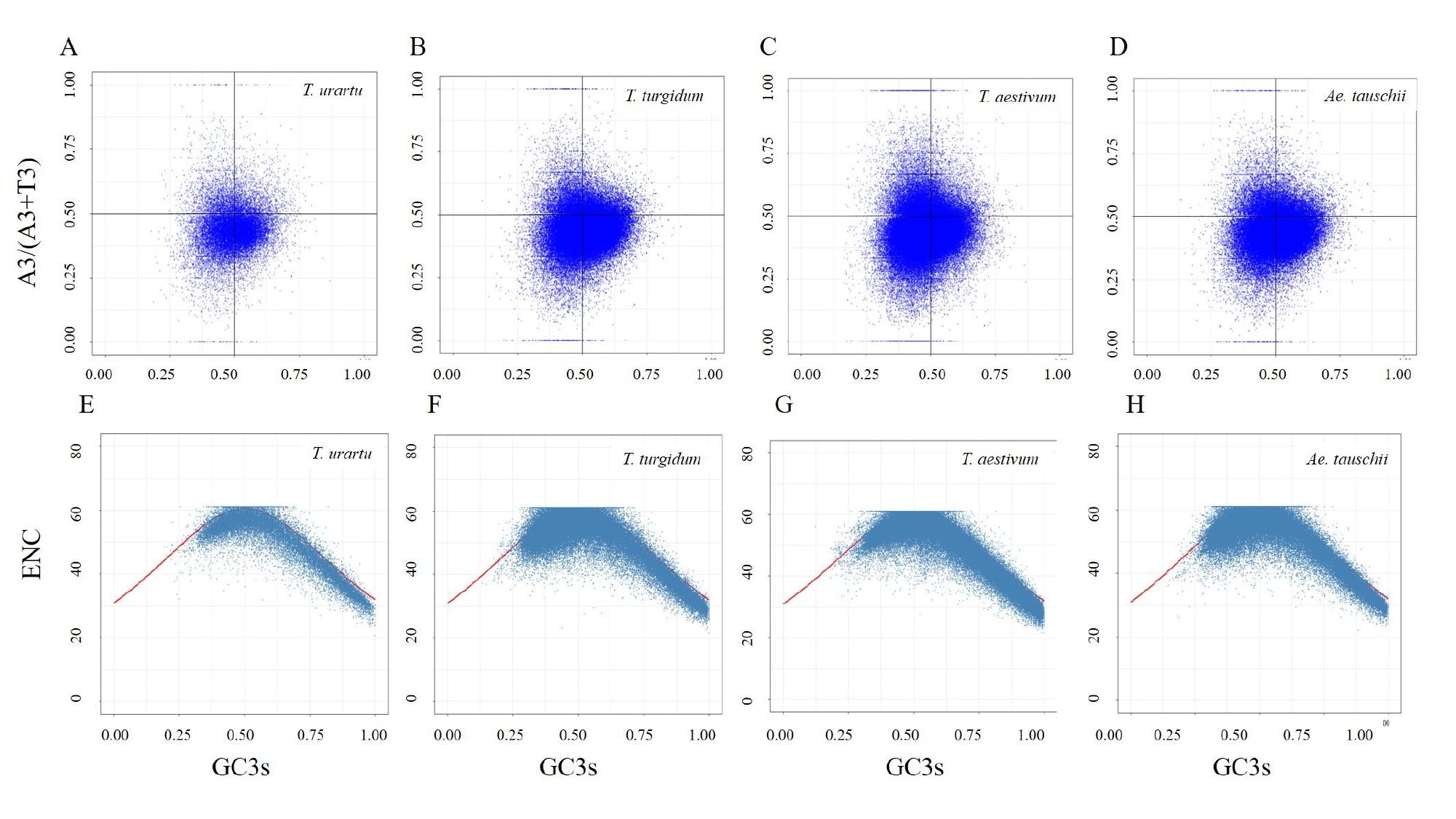

## Slide 2
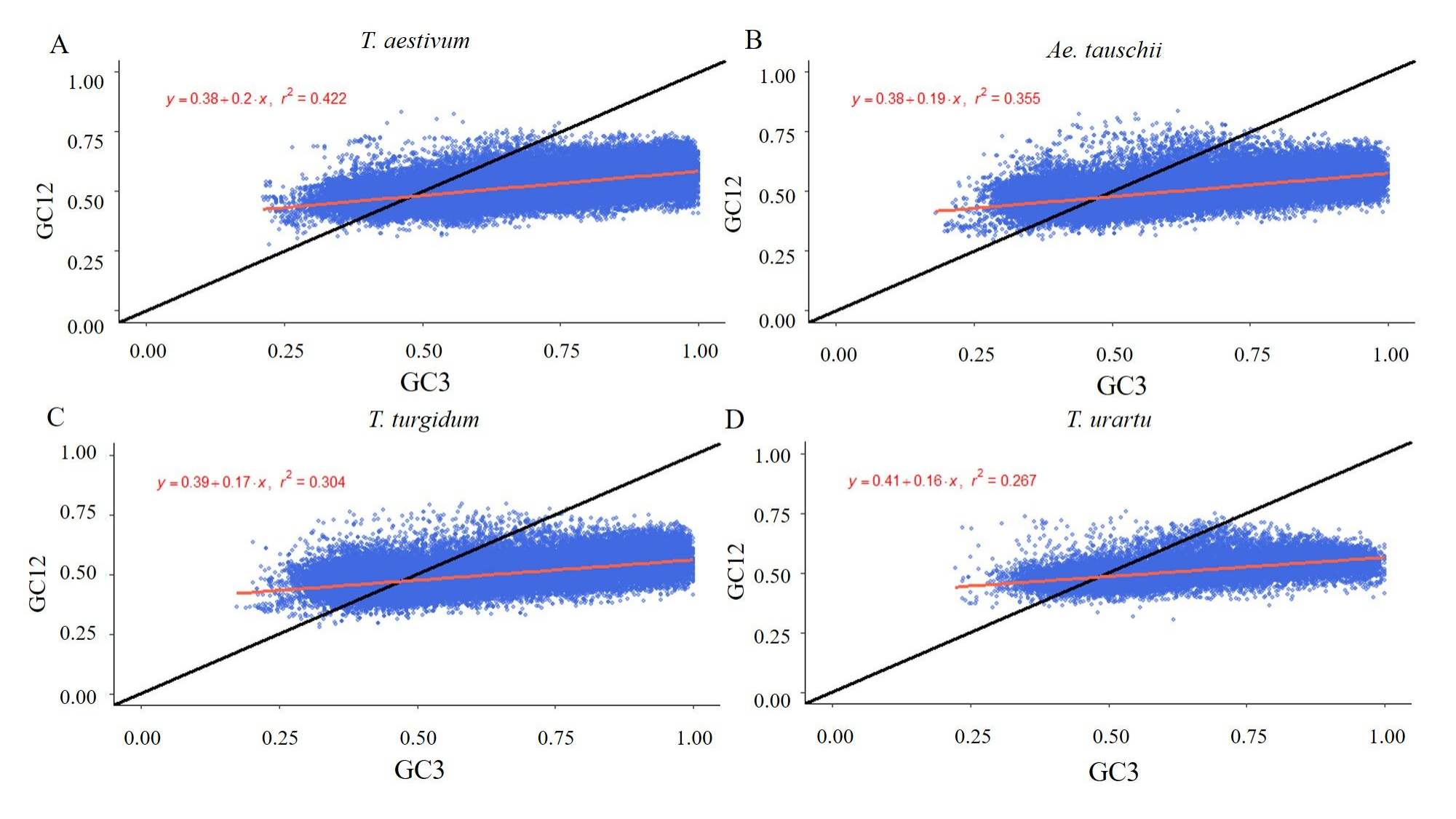

## Slide 3
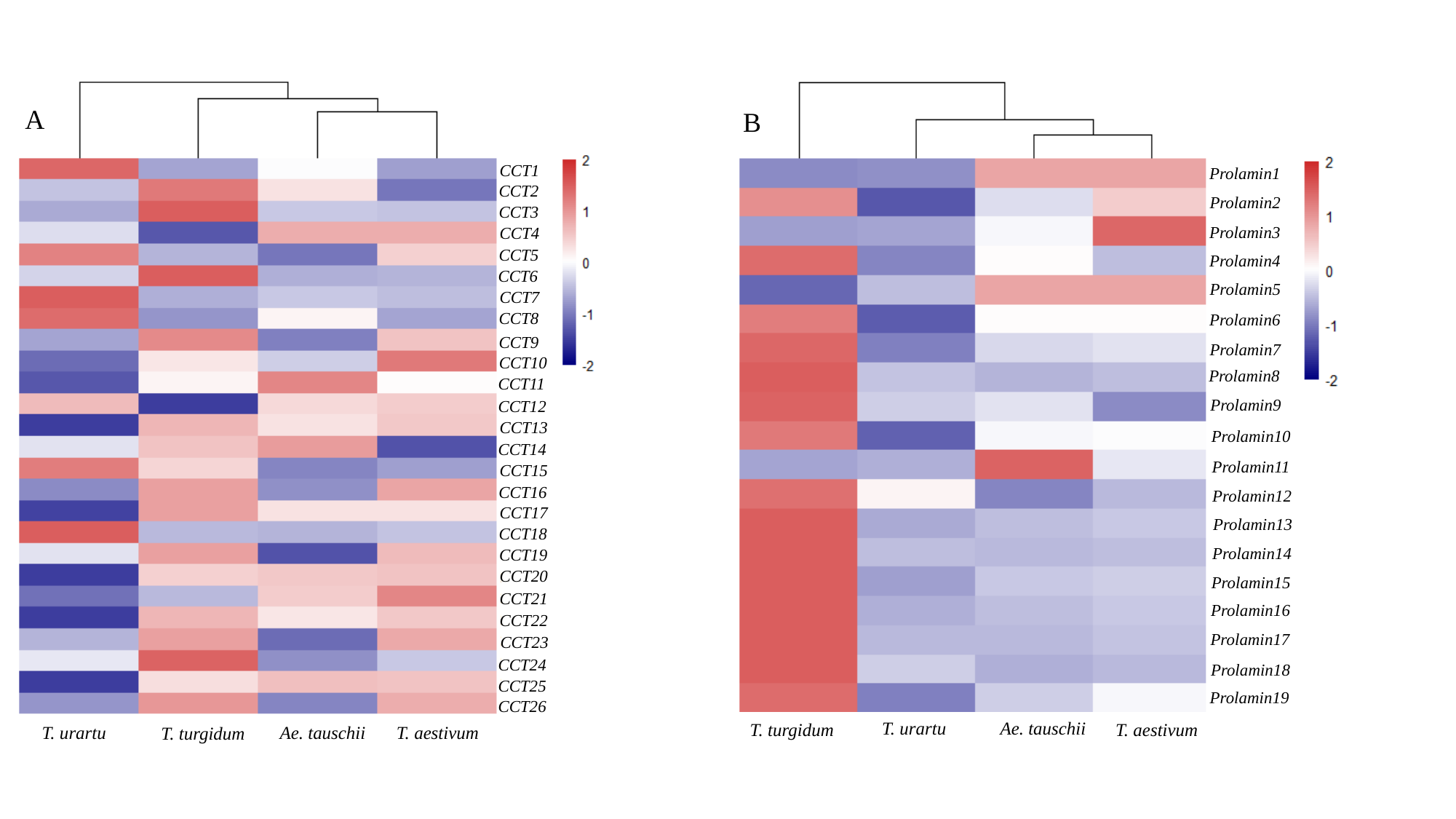

CCT1
CCT2
CCT3
CCT4
CCT5
CCT6
CCT7
CCT8
CCT9
CCT10
CCT11
CCT12
CCT13
CCT14
CCT15
CCT16
CCT17
CCT18
CCT19
CCT20
CCT21
CCT22
CCT23
CCT24
CCT25
CCT26
T. urartu
Ae. tauschii
T. aestivum
T. turgidum
A
Prolamin1
Prolamin2
Prolamin3
Prolamin4
Prolamin5
Prolamin6
Prolamin7
Prolamin8
Prolamin9
Prolamin10
Prolamin11
Prolamin12
Prolamin13
Prolamin14
Prolamin15
Prolamin16
Prolamin17
Prolamin18
Prolamin19
T. urartu
Ae. tauschii
T. aestivum
T. turgidum
B
